# Fabrication and characterization of keratin starch biocomposite film from chicken feather waste and ginger starch

**DOI:** 10.1038/s41598-021-88002-3

**Published:** 2021-04-22

**Authors:** Olarewaju M. Oluba, Chibugo F. Obi, Oghenerobor B. Akpor, Samuel I. Ojeaburu, Feyikemi D. Ogunrotimi, Adeolu A. Adediran, Makanjuola Oki

**Affiliations:** 1 Food Safety and Toxicology Research Unit, Department of Biochemistry, College of Pure and Applied Sciences, Landmark University, Omu-Aran, Kwara State Nigeria; 2 Department of Biological Sciences, Afe Babalola University, Ado-Ekiti, Ekiti State Nigeria; 3 Department of Biochemistry, Faculty of Life Sciences, University of Benin, Benin-City, Nigeria; 4 Department of Chemical Sciences, Joseph Ayo Babalola University, Ikeji-Arakeji, Osun State Nigeria; 5 Department of Mechanical Engineering, College of Engineering, Landmark University, Omu-Aran, Kwara State Nigeria

**Keywords:** Biotechnology, Environmental sciences, Materials science

## Abstract

The disposal of chicken feather through burning or burying is not environmentally compliant due to the accompanying release of greenhouse gas and underground water contamination. Thus, the transformation of this bio-waste into a bio-composite film is considered not only a sustainable strategy for disposal of this solid wastes but also an attractive alternative to developing an efficient nanostructured biomaterial from renewable bio resource. In the present study keratin extracted from chicken feather waste in combination with ginger starch were fabricated into a bio-composite film. The fabricated bio-composite films were characterized, using different analytical techniques. The physicochemical characteristics of ginger starch showed a moisture content of 33.8%, pH of 6.21, amylose and amylopectin contents of 39.1% and 60.9%, respectively. The hydration capacity of the starch was 132.2% while its gelatinization temperature was 65.7 °C. Physical attributes of the bio-composite film, such as surface smoothness and tensile strength increased significantly (*p* < 0.05) with increasing keratin content, while its transparency and solubility showed significant (*p* < 0.05) decrease with increasing keratin level. The various blends of the bio-composite films decayed by over 50% of the original mass after 12 days of complete burial in soil. Based on the results obtained in this study, the addition of keratin to starch bio-composite showed remarkable improvement in mechanical properties, such as tensile strength and surface smoothness. The bio-composite film exhibited appropriate stability in water, although future study should be carried out to evaluate its thermal stability. Nonetheless, the fabricated keratin-starch bio-composite showed desirable characteristics that could be optimized for industrial applications.

## Introduction

The lack of adequate regulatory framework guiding human activities especially in developing nations like Nigeria has inevitably led to increasing buildup of greenhouse gases leading to global warming and ultimately climate change. No doubt, the impacts of climate change is becoming more devastating with each passing year. Natural disasters, such as flooding, fire outbreaks, drought, extreme heat etc. are unforeseen consequences of climate change with devastating consequences. Global efforts aimed at reducing climate change though promising are insufficient at the moment. Thus, research efforts focusing on promoting low-cost ecofriendly approaches to meeting human needs should be encouraged^1^.

Consequent upon the growing environmental concerns on disposing packaging materials after use^2^, there is a renewed efforts aimed at promoting the use of bio-composite from renewable raw materials as alternative to single use, non-biogradable plastics. Bio-composites are evolving as a sustainable substitute to petroleum-based composites especially in food packaging applications. Hence, there is renewed research attention focusing on the fabrication of composite materials from biopolymers. Natural polymers such as proteins and polysaccharides have been reported to exhibit film-forming properties^3–5^. Unlike synthetic or hydrocarbon-based polymers, bio-based polymers are edible, ecofriendly, easily affordable and available. The development of bio-based films or coatings from renewable sources, such as polysaccharides (starch, cellulose), proteins (gelatin, keratin), lipids has attracted considerable research efforts in recent times^6–9^. Protein-based biopolymers are characterized by their safety, biodegradability and biocompatibility. Recently, Tesfaye et al.^3^ demonstrated the production of keratin-based biofilm from chicken feather waste and starch extracted from avocado seeds.

Protein processing industries including slaughter house, meat packing, leather processing plant etc. generate a huge amount of keratin containing wastes. Of these industries, the poultry slaughter industries take the lead in that approximately seven billion tons of chicken feather waste is added to the global solid waste biomass yearly^10–12^. That is, the contribution of chicken feather waste to the total solid waste is substantial. Thus, management strategies aimed at mitigating the environmental consequences of chicken feather waste is urgently required. By focusing on the physical and chemical structures and properties of feather keratin, researchers might be able to develop some economically feasible applications for the increasingly large amount of chicken feather waste generated daily^13,14^. Functional keratin has been extracted from chicken feather using various methods ranging from chemical^15,16^, enzymatic^17^ and with ionic solutions^18^. The properties of the extracted keratin has been shown to be a function of the method of extraction method. The synthesis of keratin microparticles from chicken feathers using sodium hydroxide was demonstrated by Sharma et al. and more recently, keratin solution was prepared from chicken feather for the fabrication of keratin-based bio-plastic using chemical method by Alashwal et al.^15^.

The choice of starch for an industrial product depends on its availability as well as the physicochemical properties^19^. Starch is considered a sustainable bio-resource for various industrial applications due to its abundance and low cost. More so, unlike other natural polymers such as cellulose and gums, starch can be obtained in pure form using relatively easy techniques^20^. Maize remains the main botanical source of starch accounting for about 80% starch production in the global market. However, considering the economic implication of maize utilization in terms of food security, the evaluation of new sources of starch for industrial product utilization is attracting renewed interest^21^. Ginger (*Zingiber officinale*) is a member of the Zingiberaceae. It is an herbaceous perennial root crop cultivated extensively worldwide. The underground stem or rhizome with its characteristic pungent aromatic smell is consumed as a delicacy, medicine or spice. However, with its high starch content (40–59% dry weight), it has been identified as a promising biomass for the production of starch^22,23^.

In relation to petroleum-based plastics as packaging materials in food industries, bio-based plastics exhibit lower mechanical and barrier properties but possess higher permeability to gases and water vapour^24,25^. However, the quality attributes of bio-based plastics could be considerably improved through the combination of several other biopolymers in the form of composites or blends. In recent times, there has been renewed research efforts focusing on obtaining the best combination of materials mixed for maximum efficiency. The suitability of bio-polymeric films in food packaging depends largely on the polymers as well as other materials used as additives to improve their functional properties. Starch, owing to its low-cost, availability and biodegradability is one of the most studied biopolymer with immense potential for conversion into thermoplastic starch (TPS) for food packaging. However, the high hydrophilicity of starch polymers often restricts its stability and processability as a bioresource for bio-composite film. Therefore, the combination of starch with hydrophobic biopolymers such as keratin is desirable to enhance its resistance against water as well as improved mechanical properties of the bio-composite film^25^. The present study therefore is focused on the transformation of waste keratin extracted from chicken feather waste and ginger root starch into a novel nanocomposite biofilm.

## Materials and methods

### Ethics approval

This study does not require any formal consent as it does not include any human participation or animal experimentation. All the experiment has been done in accordance with relevant institutional, national, and international guidelines/legislation.

*Chicken feather*: Chicken feather was obtained from the animal processing unit at Landmark University Commercial Farm, Omu-Aran, Kwara State, Nigeria.

*Ginger*: Ginger roots was purchased from Omu-Aran market and transported fresh in polythene bag to Biochemistry Laboratory.

*Chicken feather and keratin extraction*: Chicken feather was obtained from the animal processing unit at Landmark University Commercial Farms. The white coloured feather wastes were free of blood and grease by washing severally with detergent and then rinsed several times with tap water to remove every trace of the detergent. The washed feathers were spread on a clean dry white board to remove any extraneous tissues such as skin, intestine, beak, etc. Thereafter, the feathers were disinfected by soaking in 0.5% hypochlorite solution, sodium thiosulphate was added to neutralize the hypochlorite before being rinsed severally with distilled water before being dried to constant weight at 60 °C in a forced-air oven. The dried feathers were cut into small pieces and milled into powder, using a mechanical grinder. One thousand gram (1000 g) of the powdered feather was soaked in acetone (Sigma-Aldrich, UK) overnight, filtered and allowed to air-dry at 27 °C. Keratin was extracted from the powdered feather following a combine methods of Akpor et al.^26^ and Tesfaye et al.^3^. Fifty gram (50 g) feather powder was dissolved in 150 mL of 0.1 M sodium hydroxide solution, mixed thoroughly and then placed in an orbital shaker at 27 °C for 8 h. The resulting solution was filtered through a muslin cloth. The pH of the dissolved feather solution was adjusted to 2.4 using 10% trichloroacetic acid and the precipitated keratin filtered through a muslin cloth, washed severally in distilled water and then freeze-dried. The dried keratin was pulverized using a mechanical grinder, sieved through a 50 µm sieve and then stored at 4 °C until required for further analysis.

### Starch extraction

Starch was extracted from ginger rhizome according to the method of Afolayan et al.^23^. Freshly purchased ginger roots were neatly peeled, washed with copious water, sliced into small pieces and soaked in 1% sodium metabisulphite solution to prevent oxidative browning. Afterward, the chopped ginger roots were milled into a slurry using an electric blender. The starch slurry was then dispersed in a large volume of 1% sodium metabisulphite solution before been filtered using muslin cloth. The obtained filtrate was centrifuged for 15 min at 3000 rpm after which the supernatant was carefully decanted and the mucilage scraped off. The process was repeated trice until the starch was obtained in a pure form. The extracted starch was oven-dried to constant weight at 30 °C, weighed and then stored at 4 °C, until required for further analysis.

### Preparation of the keratin-starch bio-composite film

In the preparation of the keratin-starch bio-composite film, the method described by Tesfaye et al.^3^ was followed with little modifications. Briefly, a 5% starch solution was prepared and gelatinized by heating on an electric heater at 70 °C under constant stirring. Keratin solution was made by adding 5 g keratin powder in 100 mL of 0.1 M NaOH and heated in an electric heater at 70 °C for 20 min with constant stirring. Different blends of starch and keratin solutions were then prepared as follow: 20:0 (control), 20:1, 20:3 and 20:5 (starch:keratin ^v^/_v_). To each starch-keratin blend 1 mL of glycerol was added as plasticizer, after which the mixture was heated at 70 °C on an electric heater for 10 min, with vigorous stirring before being poured into a glass petri dish and oven-dried at 50 °C for 24 h. The resulting film was carefully peeled and kept in a cool dried carton at 25 °C before further analysis.

### Physicochemical characterization of ginger starch

#### Swelling power determination

The swelling power of the starch sample was determined using the method of Afolayan et al.^23^. Ginger starch (0.1 g) was added to 10 ml of distilled water and heated at 50 °C for 30 min in a water bath with continuous shaking. The resulting slurry was centrifuged at 2000 rpm for 15 min after which the supernatant was carefully decanted and the resulting starch paste weighed. Thereafter, the swelling power was estimated from Eq. (1):

1 $$Swelling\,power = \frac{Weight\,of\,starch\,paste}{{Weight\,of\,dry\,starch\,sample}} \times 100$$

The swelling power of the starch sample was estimated over a temperature range of 50–100 °C.

#### Solubility index determination

The swelling power of the starch sample was determined using the method of Afolayan et al.^23^. A starch solution comprising of 0.5 g dry starch sample dissolved in 10 mL distilled water and heated at 50 °C over a water bath for 30 min with vigorous shaking was prepared. The starch solution was then centrifuged at 2000 rpm for 10 min and the supernatant carefully decanted. The resulting starch paste was weighed and the solubility index was estimated using Eq. (2):

2 $$Solubility\,index = \frac{Weight\,of\,starch\,paste }{{Weight\,of\,dry\,starch\,sample}} \times 100$$

The solubility index of the starch sample was evaluated over a temperature range of 50–100 °C.

#### pH determination

The pH was determined using a British Standard Institution (BSI 757) as described by Taheri et al.^27^. A10% (^w^/_v_) starch dispersion was prepared with continuous shaking in a water bath at 50 °C for 5 min. Having allowed the starch gel to cool to about 25 °C, the pH determined using a pH meter with a glass electrode after standardizing with 4 and 7 pH buffers.

#### Gelatinization temperature determination

The gelatinization temperature was determined following the method described by Nwokocha et al.^28^. One gram (1 g) starch sample was dispersed in 10 mL distilled water and stirred vigorously. The starch solution was placed on a hot plate and heated with continuous stirring. With the aid of a suspended thermometer, the gelatinization temperature was then estimated.

#### FTIR analysis

In order to ascertain the effect of sodium hydroxide used for the hydrolysis of chicken feather on the isolated keratin, FTIR analysis of both raw feather powder and the keratin obtained in this study. This was done using an FTIR Spectrometer (Bruker OPTIK, Germany).

### Physicochemical characterization of the keratin-starch bio-composite film

#### Thickness measurement

The bio-composite film thickness was estimated with the aid of a digital MDC-MX 923 µm (Mitutoyo, Japan). For each bio-composite film, 10 random points were sampled and measurements were taken and the mean values calculated.

#### Transparency test

The degree of transparency or opaqueness of the bio-composite film with the aid of a UV–visible spectrophotometer (Jenway 7305, UK) at 560 nm. The measurement was done following Santacruz et al.^29^ method.

#### Moisture content determination

The moisture content of the bio-composite film was determined by drying the bio-composite film to constant weight in a forced air oven at 100 °C. The moisture content was then calculated using Eq. (3):

3 $$Moisture\,content\,(\% ) = \frac{W1 - W2}{{W1}} \times 100$$

where $W1$, Initial weight of the biocomposte film, $W2$, weight of the bio-composite film after drying.

#### Tensile strength analysis

The tensile strength of the bio-composite film was determined according to the method of Rahman and Jamalulail^30^, with the aid of a Universal Testing Machine (Hounsfield Series S, UK) according to ASTM standard D882. In measuring the tensile strength, a 1.0 kN load was applied at 10 mm/mim constant at 27 °C. From the data obtained, tensile strength and elongation at break were calculated.

#### Solubility test

The solubility of the keratin-starch bio-composite film in water was determined according to the method of Pavin et al.^31^. A weighed amount of the keratin-starch bio-composite films was completely immersed in water for a period of 12 da and at every three-day interval, the bio-composite film was exhumed and weighed.

#### Degradability test

The degradability test of the keratin-starch bio-composite films in soil was studied, following the method of Pavin et al.^31^. In this study, a weighed amount of the keratin-starch bio-composite films was vertically buried at 3–5 cm depth inside the soil to ensure aerobic degradation conditions at 25 ± 2 °C and 35–50% relative humidity for a period of 12 days and at every three-day interval, the bio-composite film was exhumed, washed with water, dried to constant weight and weighed.

#### Morphological analysis of the bio-composite films

The morphological analysis of keratin, ginger starch, and keratin-starch bio-composite film was carried out with a field emission gun scanning electron microscope (SEM) (Zeiss, Germany). The sample was placed on a gold coated stub with the aid of a diode sputter coater. The crystalline phase evaluation was carried out with the aid of a benchtop XRD analyzer (ARL EQUINOX 1000, Thermo Fischer, UK), equipped with a diffracted beam monochromator and a copper target X-ray tube operated at 40 kV and 30 mA.

### Statistical analysis

The experimental set up was carried out in triplicate and the results expressed as mean ± SD. The results were statistically analyzed using two-way analysis of variance (ANOVA) followed by Turkey multiple range test. Charts were drawn using GraphPad prism 8.0 software (GraphPad Software Inc., San Diego, California). Significant level was set at *P* < 0.05.

## Results

### Physicochemical characteristics of ginger starch

**Figure 1:**
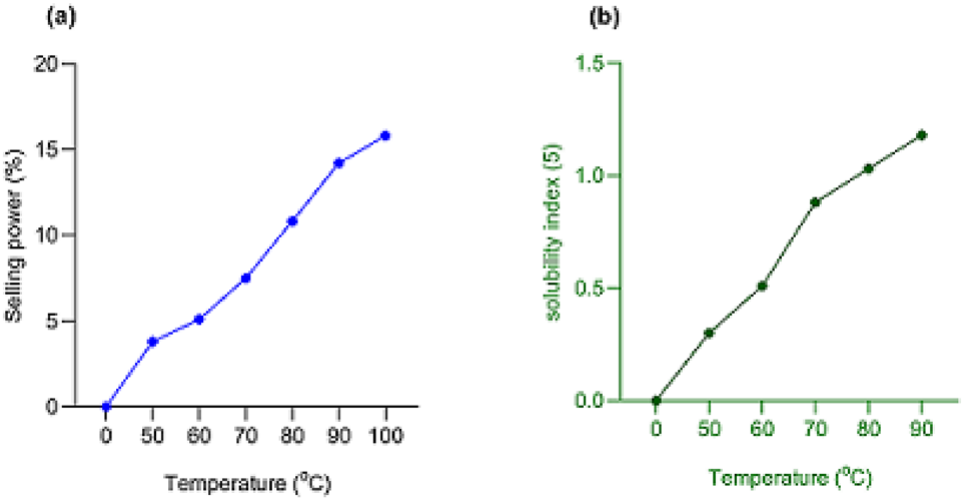
Swelling power and solubility index of ginger starch. Results are means ± SD of triplicate determinations. Charts were drawn using GraphPad prism 8.0 software (GraphPad Software Inc., San Diego, California), https://www.graphpad.com/scientific-software/prism.

The physicochemical characteristics of ginger starch showed a moisture content of 33.8%, pH of 6.2, amylose and amylopectin contents of 39.1% and 60.9%, respectively. The hydration capacity of the starch was 132.2% while its gelatinization temperature was 65.7 °C (Table 1). Ginger starch showed a progress increase in swelling capacity (Fig. 1a) and solubility index (Fig. 1b) with increasing temperature.

**Table 1:** Physicochemical characteristics of ginger starch.

Moisture (%)	pH	Amylose (%)	Hydration capacity (%)	Gelatinization temperature (^o^C)
33.8 ± 0.44	6.2 ± 0.1	39.1 ± 1.6	132.2 ± 18.1	65.7 ± 3.2

Results are means ± SD of triplicate determinations.

### FTIR analysis

**Figure 2:**
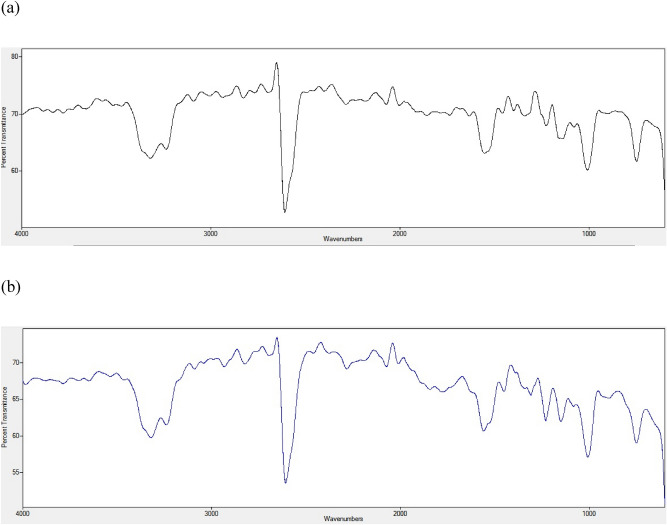
FTIR of (**a**) raw chicken feather and (**b**) keratin obtained following NaOH hydrolysis of chicken feather.

The FTIR spectra of both raw chicken feather (Fig. 2a) and the obtained keratin from hydrolyzed chicken feather (Fig. 2b) showed similar absorption peak at 3284.7 cm^−1^ assigned to O–H stretching of alcohol. The amide bond, C=O stretching vibration showed absorption peak at 1680 cm^−1^. The C–H stretching vibration was revealed at 1550 cm^−1^ while the absorption peak at 1280 cm^−1^ corresponds to C-N stretching. In addition, the raw chicken feather showed S–H stretching of thiol at 2574.6 cm^−1^ which appeared to have been suppressed in keratin.

### Physical attributes of keratin-starch bio-composite film

**Figure 3:**
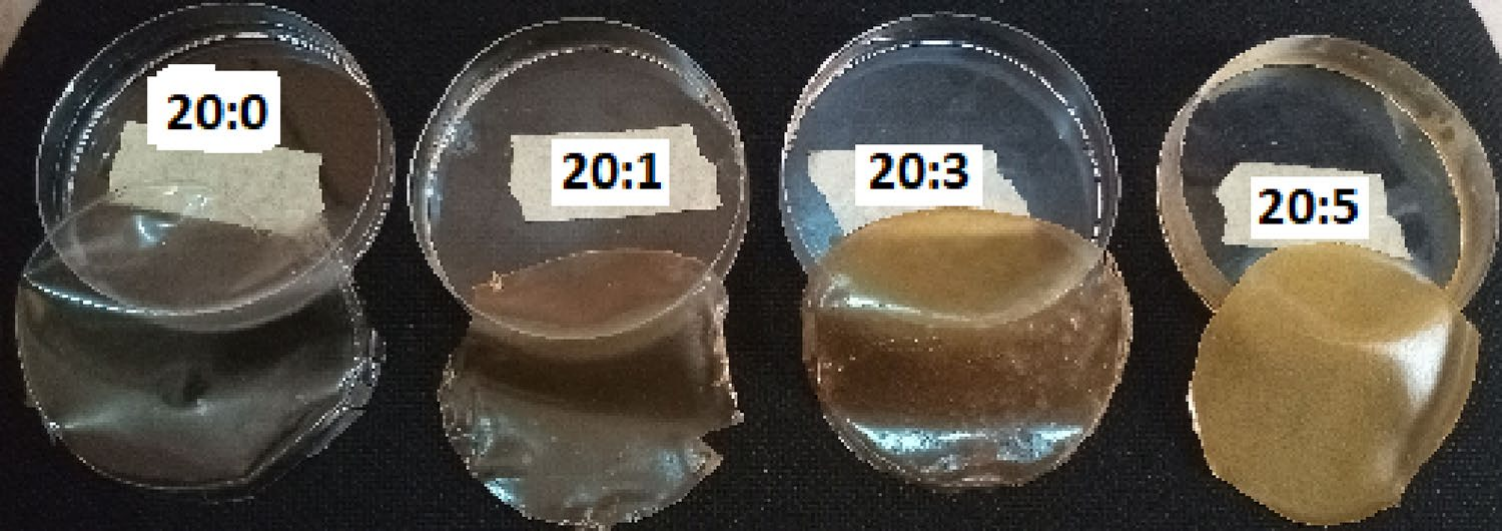
Images bio-composite films made with different blends of keratin and starch solutions. Note: 20:0, 20:1, 20:3, and 20:5 = starch/keratin (^v^/_v_).

The fabricated bio-composite films obtained from starch, and a combination of starch and different volumes of keratin with glycerol as plasticizer is shown in Fig. 3. Keratin containing bio-composites (20:1, 20:3, and 20:5) had significantly lower moisture content, when compared to the starch only (20:0) bio-film. The moisture content of the keratin-based bio-composite (20:1, 20:3, and 20:5) decreased with increasing keratin content. Bio-composites containing 1 and 3 volume of keratin had similar moisture level which was significantly (*p* < 0.05) higher than 20:5 bio-composite (Table 2). On the contrary, the thickness of the bio-composites increased proportionately with increasing keratin content. The 20:3 and 20:5 keratin-containing bio-composites were significantly thicker compared to 20:1 bio-composite and 20:0 bio-films. Film transparency follows a similar trend with moisture content. Opaqueness increases with increase in keratin-level in the bio-composites (Table 2). The tensile strength and elongation at break of the bio-composite films increased significantly (*p* < 0.05) with increasing keratin concentration in the film.

**Table 2:** Characteristics of the keratin-starch bio-composite films.

Characteristics	20:0	20:1	20:3	20:5
Moisture (%)	19.0 ± 0.1^c^	13.0 ± 0.2^b^	11.7 ± 0.2^b^	5.5 ± 0.2^a^
Thickness (mm)	0.18 ± 0.02^a^	0.23 ± 0.03^b^	0.35 ± 0.03^c^	0.35 ± 0.04^c^
Transparency (%T)	76.83 ± 0.3^d^	52.60 ± 0.2^c^	33.20 ± 0.1^b^	18.03 ± 0.2^a^
Tensile strength (MPa)	8.3 ± 0.2^a^	9.2 ± 0.1^a^	11.5 ± 0.3^b^	13.8 ± 0.2^c^
Elongation at break (%)	19.7 ± 0.1^a^	20.5 ± 0.3^a^	28.1 ± 0.3^b^	33.3 ± 0^c^

Results are means ± SD of triplicate determinations. Values in the same row carrying different alphabets are significant (*p* < 0.05). Note: 20:0, 20:1, 20:3, and 20:5 = starch/keratin (^v^/_v_).

### Solubility test

**Figure 4:**
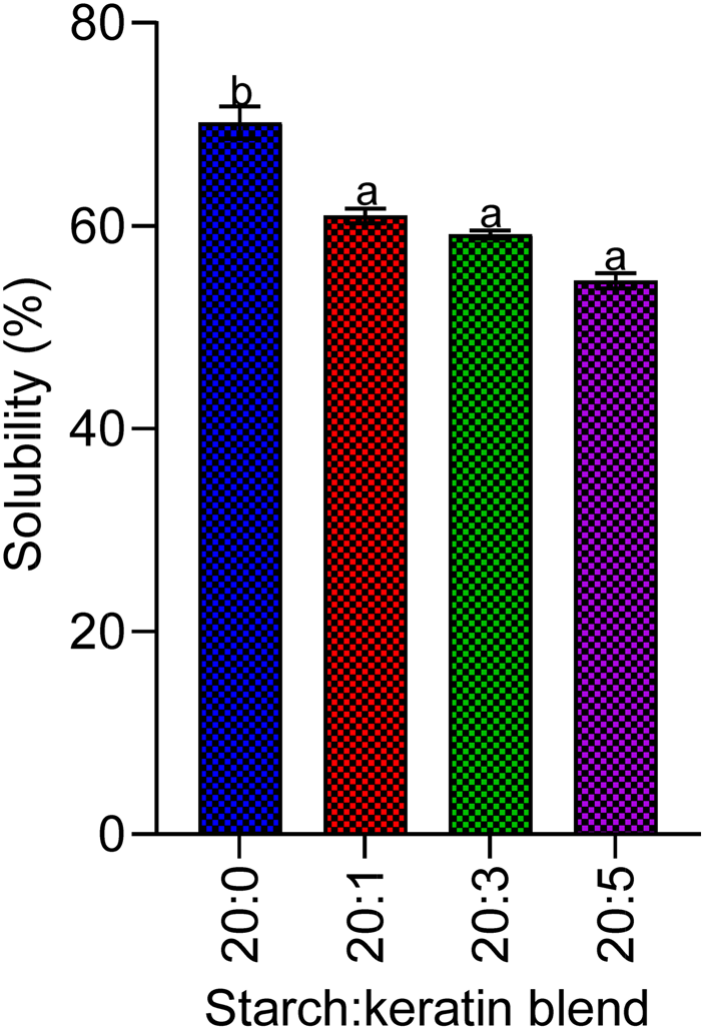
Solubility of the starch:keratin biocomposite film in distilled water. Results are means ± SD of triplicate determinations. Bars carrying different alphabets are significant (*p* < 0.05). Note: 20:0, 20:1, 20:3, and 20:5 = starch/keratin (^v^/_v_). Chart was drawn using GraphPad prism 8.0 software (GraphPad Software Inc., San Diego, California), https://www.graphpad.com/scientific-software/prism.

Keratin containing bio-composites (20:1, 20:3, and 20:5) had significantly lower solubility in water, when compared to the starch only (20:0) bio-film. The solubility of the keratin-based bio-composite (20:1, 20:3, and 20:5) decreased with increasing keratin content though the observed decreases were insignificant (*p* > 0.05) (Fig. 4).

### Degradability test

**Figure 5:**
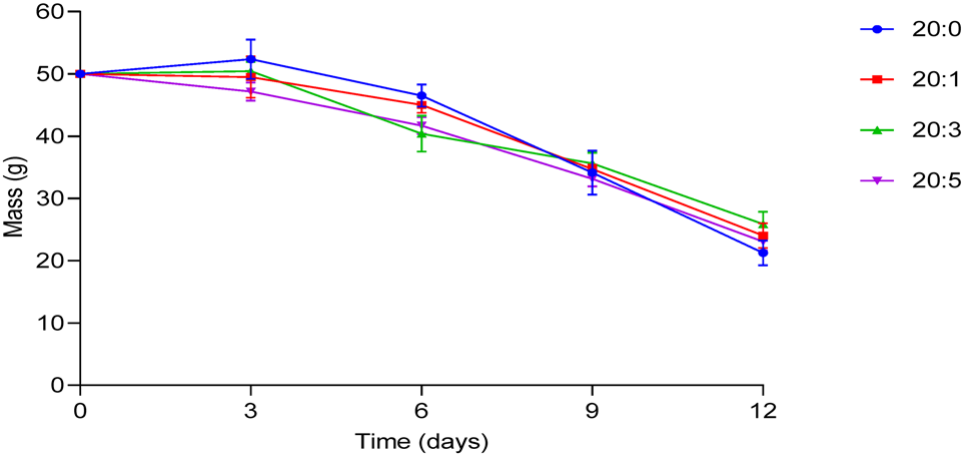
Degradation curve of the starch:keratin bio-composite films from a 12-day burial in soil. Results are means ± SD of triplicate determinations. Note: 20:0, 20:1, 20:3, and 20:5 = starch/keratin (^v^/_v_). Chart was drawn using GraphPad prism 8.0 software (GraphPad Software Inc., San Diego, California), https://www.graphpad.com/scientific-software/prism.

The various blends of the bio-composite films decayed by over 50% of the original mass after 12 d of complete burial in the soil. No appreciable difference was however observed in the degree of degradability between the various blends on day 12 (Fig. 5).

### Structural analysis

**Figure 6:**
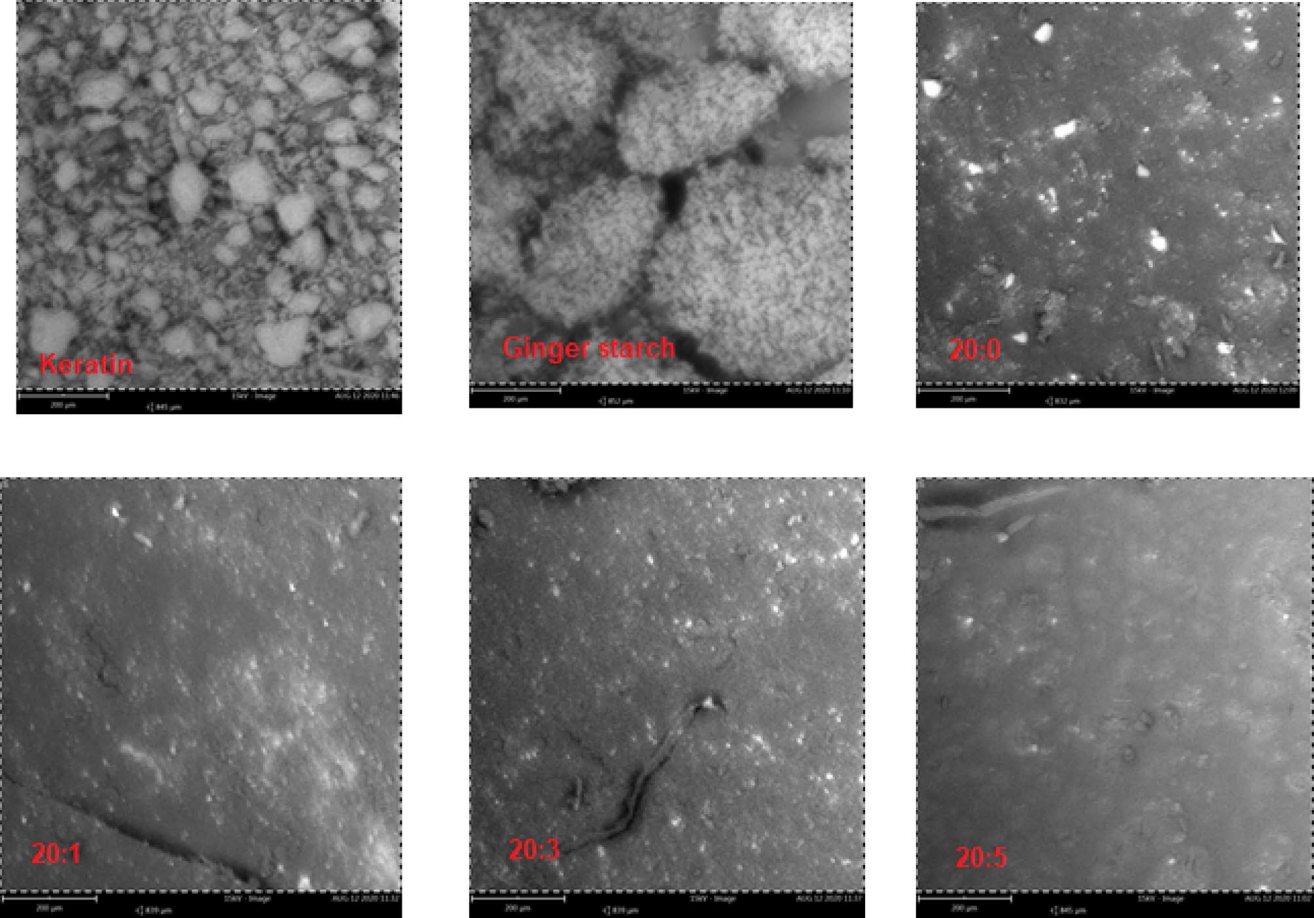
SEM images of keratin, ginger starch, and bio-composite films made of difference blends of starch and keratin solutions. Note: 20:0, 20:1, 20:3, and 20:5 = starch/keratin (^v^/_v_).

**Figure 7:**
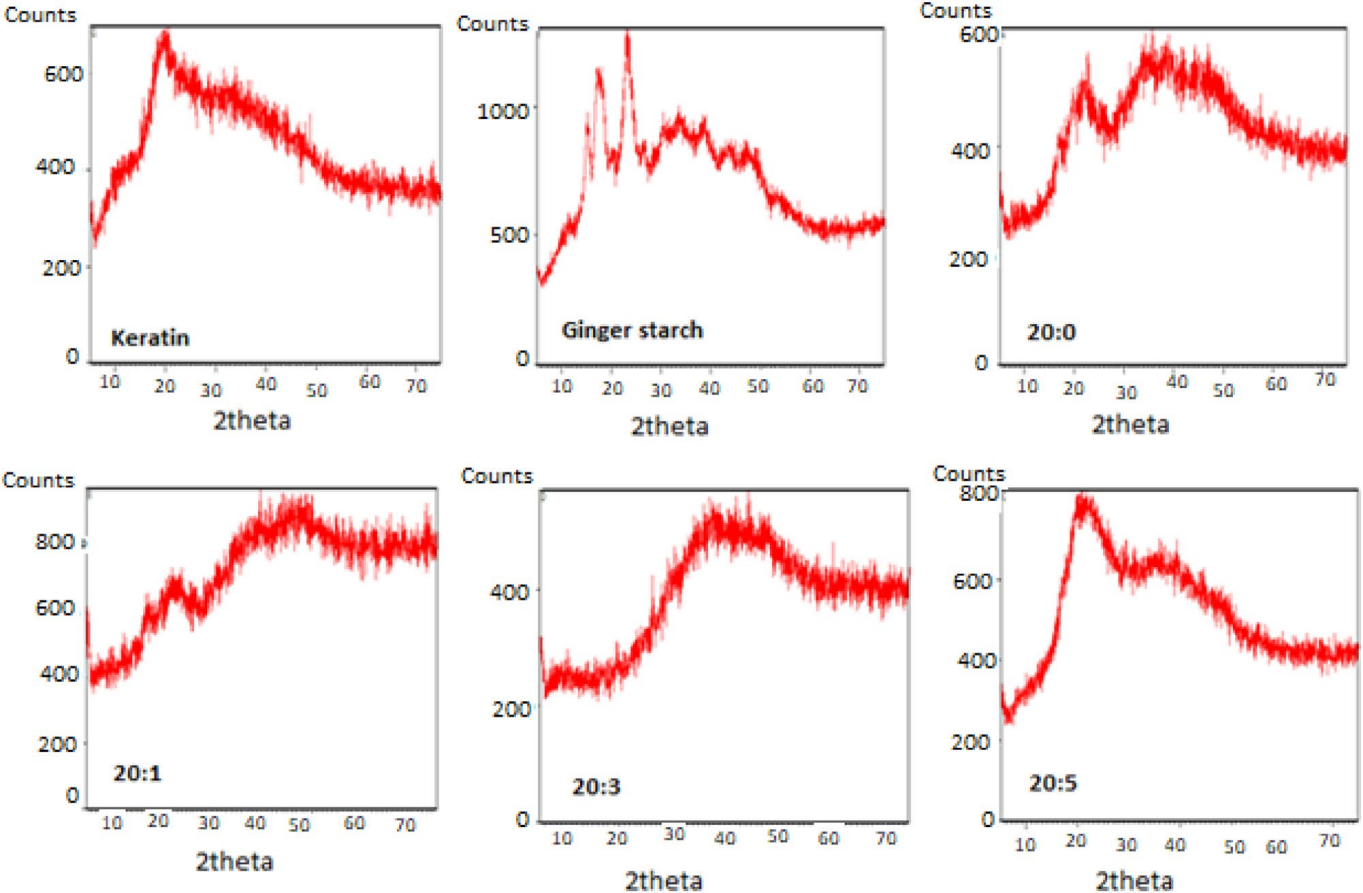
XRD spectra of keratin, ginger starch, and biocomposite films made of difference blends of starch and keratin solutions. Note: 20:0, 20:1, 20:3, and 20:5 = starch/keratin (^v^/_v_).

Scanning electron micrographs of keratin, ginger starch, and bio-composite films obtained from graded addition of keratin to starch solution are presented in Fig. 6. The SEM images showed distinct variation characteristic of their respective composition. The roughness of the bio-composite surface was observed to decreases with increasing keratin concentration. X-ray diffractogram of ginger starch showed distinct characteristic crystalline peaks, which was observed to disappear with increasing keratin content in the keratin-starch bio-composite films (Fig. 7).

## Discussion

The percentage moisture content of ginger starch as demonstrated in this study is considerably high and poses a great threat in terms of its shelf life^32^. The stability and storage of food products depend greatly on their moisture content. A moisture content of 10–12% is recommended for commercial starches and starch with a below 10% moisture level is specified for the production of low-density bio-composite film^33,34^. A pH of 6.2 obtained for ginger starch in this study agrees closely to the value (6.2) reported by Praveen et al.^35^. The amylose content (39.1%) of ginger starch as revealed in this study is high compared to values obtained for wheat (25.6%)^36^; potato (29.3%), cassava (23.7%), and *Dioscorea* (26.3%)^37^. However, our result agrees closely with that obtained for ginger (34.0%) by Braga et al.^38^. Turmeric had been demonstrated to contain amylose content as high as 48%^38^. The amylose content of starch contributes significantly to its physicochemical and functional characteristics. Studies have shown that the amount of amylose present within a particular crop could vary even within the same botanical species due geographical and cultural variations^39^. Amylose unlike amylopectin is anhydrous hence impede the swelling and solubility of starch granules. The anhydrous nature of amylose offers excellent advantage in industrial application of starch in the fabrication of very strong, colourless bio-composite films^40^. Starches with amylose content above 30% are known to show a slight deformation in their granule appearance. The interactions within the polymeric linkages of starch determine its solubility and swelling capacity^41^. These interactions in turn is a function of the ratio of amylose to amylopectin^42^. The lower the amylose to amylopectin ratio, the greater is the swelling capacity of starch^39^. Starches such as ginger starch with high amylose content as demonstrated in this study usually exhibit low swelling and solubility capacity. The high amylose of ginger starch (> 30%) contributes significantly to the mechanical properties of bio-composite films. The considerably high amylose content of ginger starch in this study also contributes to its relatively high gelatinization temperature. Studies have shown that potato starch, wheat starch, and cassava starch with low amylose level exhibit a relatively low gelatinization temperatures of 57–58°C^43^. It is often argued that the longer branch chains in high-amylose starches is responsible for their characteristically high gelatinization temperatures. Gelatinization is a requisite process which disrupts the crystalline structure of starch as a means of facilitating good cross-linking reactions in bio-composite formation.

The present study demonstrated the successful fabrication of thin, homogenous, yellow flexible films from varying combinations of ginger starch and chicken feather keratin. The colour variation from white to yellow as a function of increasing keratin content led to reduction in the degree of transparency of the bio-composite films. This gives an indication that keratin reduces the transparency of bio-composite film owing to the increasing solid particles in the filmogenic solution. Composites with high degree of opacity have been shown to exhibit high UV-barrier property, a desirable attribute in food packaging materials. This observation concurs with the report of Tesfaye et al.^3^ on the production of bio-composite film from avocado pear kernel and keratin. The inclusion of keratin in the biofilms was also observed to enhance the toughness, in terms of tensile strength, of the films in addition to glycerol. This could be due to the formation of intermolecular bonds/interactions between the hydroxyl groups present in starch and the functional groups (carboxyl and amino groups) present in keratin amino acids. Thus, the keratin-based biofilms had higher tensile strength and exhibited higher elongation at break than the one containing ginger starch and glycerol only.

The moisture level in the various blends of keratin-starch bio-composite films ranged between 5.5 and 19.0%. The moisture absorption capacity of bio-composites is of great significance with regard to their processing, packaging, transportation, and shelve life. During processing, moisture content of products could affect the intra and intermolecular associations within and/or between molecules present in such products thereby contributing its final mass or volume as well impact on its durability. Products with high moisture content most often tend to be heavier hence high transportation cost and are also highly susceptible to rapid deterioration^44,45^. The inclusion of keratin in the fabricated bio-composites in this study led to remarkable reduction in moisture content. This suggests enhanced shelf life, as a result of low microbial activity, of the bio-composite film and reduced cost implication with regard to transportation. The interaction affinity of a biofilm with an aqueous environment such as obtainable within a cellular milieu is determined by its solubility. Though the utilization of a bio-composite film in food packaging only requires partial solubility in water in order to not to compromise the integrity of packed products, the solubility of certain products before consumption is beneficial. From the present study, it was observed that keratin inclusion resulted in remarkable decrease in solubility. The reduction in solubility as a consequence of keratin could be attributed to the presence of hydrophobic amino acids within the keratin matrix^46^. It is our submission that the potential reduction in solubility of bio-composite film as a result of keratin could be beneficial in contributing to their stability.

The results of the SEM images evidently showed that the morphological appearance of keratin-starch bio-composites greatly depends on their chemical composition. Film attributes, such as surface morphology and texture are influenced by the ratio of the keratin and starch solutions. Contrary to reports by some authors, we observed that the degree of smoothness of the keratin-starch bio-composite film depends on the keratin content and not starch. From the data obtained from the X-ray difractograms, it could be observed that the XRD patterns of ginger starch showing different crystallinity peaks, completely disappeared, especially from the 20:3 keratin-starch bio-composite film. This is indicative of a perfect dispersion of the starch granules with the keratin fibres showing the extent of intercalation or cross linking in the bio-composite film.

Data obtained from the degradation of the bio-composite fil in soil was impressive. The loss of over 50% mass observed in the keratin-starch bio-composite film after 12 ds is significant, given the challenge in chicken feather waste disposal. The soil contain a myriad of microflora consisting of bacteria and fungi. This microbial population produce different types of keratinases that act synergistically together in the degradation process^47^.

## Conclusion

These study demonstrated the successful fabrication of a keratin-starch bio-composite film from chicken feather wastes and ginger starch. The addition of keratin to starch-based composite was observed to enhance the suitability of bio-composite for food packaging through improved quality attributes including reductions in moisture content, opacity or transparency, as well water solubility of the bio-composite films. It also showed desirable mechanical properties such as high tensile strength and elongation at break, thus contributing to its stability. The remarkable degradability of the bio-composite film is a testament of its environmental friendliness. Overall, the keratin-starch bio-composite showed comparable qualities to the non- degradable polymers. Therefore, it could offer a viable, eco-friendly and sustainable solution to solid waste accumulation in the environment.

## Data Availability

All data generated and analyzed during the current study are available with the corresponding author upon reasonable request.
